# Effect of Three Bean Species on the Development and Reproduction of a Population of the Parasltold, *Encarsia bimaculata,* on the Whitefly, *Bemisia tabaci*

**DOI:** 10.1673/031.010.2801

**Published:** 2010-04-03

**Authors:** Augustine Mansaray, Abu James Sundufu

**Affiliations:** ^1^ Institute of Agricultural Research, Njala, Sierra Leone; ^2^ Department of Biological Sciences, School of Environmental Sciences, Njala University, Sierra Leone

**Keywords:** parasitism, fecundity, emergence, longevity

## Abstract

Developmental time, parasitism, emergence, longevity, fecundity and demographic parameters of population of *Encarsia bimaculata* Heraty and Polaszek (Hymenoptera: Aphelinidae), a parasitoid attacking *Bemisia tabaci* (biotype B) (Gennadius) (Homoptera: Aleyrodidae) infesting soybean, *Glyine max* L. (Merr), cowpea, *Vigna unguiulata* L. and garden bean, *Phaseolus vulgaris L.* (Fabeles: Fabaceae) were quantified and compared. *Encarsia bimaculata* was able to complete its life cycle independent of the *B. tabaci* instar parasitized. However, parasitoid development was significantly slower when first (19 d), second (15 d) instars or pharate adults (14 d) were parasitized compared to the third (13 d) or fourth (13 d) instars. Consequently, percent parasitism was higher when the third (51 %) or fourth (46 %) instars were parasitized compared to the first (22 %), second (25 %) instars or pharate adults (36 %) of *B. tabaci.* Similarly, percent parasitoid emergence was significantly higher when third (83 %) or fourth (76 %) instars were parasitized compared to when the first (34 %), second (64 %) or pharate adults (54 %) were parasitized. Host plant species significantly influenced egg to adult developmental time, percent parasitism and the day on which *E. bimaculata* nymphs hatching from eggs was first observed. More nymphs were parasitized on cowpea (40 %) followed by garden bean (36 %) and soybean (32 %), while percent hatching was significantly higher on soybean (76 %) followed by cowpea (68 %) and garden bean (42 %). Adult parasitoid females lived an average of 6.7 d on soybean, 7.6 d on cowpea and 7.2 d on garden bean and laid a lifetime average of 27 eggs on soybean, 31 eggs on cowpea and 30 eggs on garden bean. The daily mean fecundity of *E. bimaculata* was not significantly different on the three bean species. Life table parameters showed that the net reproductive rate (R_o_) was 14.50, generation time (T_c_) was 17.16, intrinsic rate of natural increase (*r_m_*) was 0.16, finite rate of growth (λ) was 1.17 and doubling time (T_d_) was 4.44 for parasitoids on soybean. On cowpea, R_o_ was 15.32, T_c_ was 18.59, *r_m_* was 0.15, λ was 1.16 and T_d_ was 4.72, while, on garden bean, R_o_ was 8.95, T_c_ was 19.28, *r_m_* was 0.11, λ was 1.12 and T_d_ was 6.08. Given these life table parameters, higher population build up of the parasitoid will be expected on cowpea and soybean, respectively, compared to garden bean. Thus, for an effective augmentative release program involving *E. bimaculata* for the control of *B. tabaci,* it is important to take into consideration both the host stage of *B. tabaci* and the nature of the host plant on which it is developing.

## Introduction

The sweet potato whitefly *Bemisia tabaci* (Gennadius) (Hemiptera: Aleyrodidae) biotype B, is a major pest and plant virus vector attacking a wide variety of food crops worldwide (Perring 2001). The expulsive increase in *B. tabaci* population has been attributed in part, to the heavy application of pesticides to control it that in turn has resulted in the development of pesticide resistance and in the decline of the whitefly's natural enemies (Van Lenteren 2000). Therefore, it became important to promote the use of effective biological control agents that can efficiently control the pest, are safe to the environment and are acceptable to the farmers and greenhouse growers (Heinz 1995).

Parasitoids of the genera, *Amitus, Eretmocerus* and *Encarsia* are among the most important natural enemies of the *B. tabaci* complex (Cock 1996, Hoelmer 1995). Heinz and Parella (1998) showed that the percentage of parasitoids successfully developing to adults was greater for *Encarsia formosa* than for *Eretmocerus* sp., independent of the host plant. Collection records and descriptions of parasitism of *Bemisia* spp. worldwide indicate differences in guild structure and parasitism intensity depending on the species of host plant sampled and host plant age and size (Hu and Vinson 2000; Hu *et al.* 2002).

A study on the effects of two different host plant species, each infested with *B. tabaci,* on the fitness of two populations of *Eretmocerus* sp. was conducted by Powell and Bellows (1992). The study showed significant differences in preimaginal (development during the egg stage) developmental rate, survival and fertility between host plant species. Also, Liu and Stansly (1996) described the effect of *B. tabaci* age at the time of parasitization on the number of egg oviposited, egg to adult development and survivorship of *Encarsia pargandiella.*

The objectives of this study were to compare reproduction of *Encarsia bimacalata* attacking various stages of *B. tabaci* infesting soybean, *Glycine max* L. (Merr), cowpea, *Vigna unguiculata* L. and garden bean, *Phaseolus vulgaris* L. (Fabeles: Fabaceae) and to see how best host plant resistance and biological control can be integrated to suppress whitefly population.

## Materials and Methods

### Plant materials

Seeds of *G. max, P. vulgaris* and V. *unguiulata* were obtained from Guangdong Agricultural Institute in Guangzhou, South China. The seeds were placed in Petri dishes with water to initiate germination. The partially germinated seeds were grown individually in 12 cm diameter plastic pots and used in the experiment at the 4–6 leaf stage. These pots were placed into cages (60 × 60 × 60 cm).

### Host and parasitoid culture

Adult *B. tabaci* were collected from cucumber plants in the field and released into cages (60 cm × 60 cm × 60 cm) containing cucumber as the source host. These cages were placed in a temperature controlled room maintained at 25° C with a relative humidity of 70 % under a photoperiod of 14:10 (L:D).

*Encarsia bimaculata* cultures were established by collecting parasite wasp pupae from fields located in South China Agricultural University. These were brought to the laboratory and placed into Petri dishes with 10% honey solution as food following eclosion. Adult parasitoids were released on caged cucumber, *Cucumis sativus* L. (Malvales: Malvaceae) plants infested with with *B. tabaci* nymphs. Pupae of *E. bimaculata* turn black inside of parasitized *B. tabaci.* These black pupae were sexed as female or male by using the criterion that females produce two meconial pellets while males produce four. Virgin female parasitoids were obtained by confining female pupae in vials. Upon emergence, female parasitoids were placed on detached cucumber leaf with abundant whitefly nymphs for approximately six hours. The detached cucumber leaf was confined in a Petri dish, and the leaf petiole was covered with moist cotton wool to retain leaf turgidity. Parasitoids obtained sufficient nutrients for egg lying by feeding on honeydew and the body fluid of whitefly nymphs, which oozed out of ovipositor punctures. Mated female parasitoids were obtained by confining a newly emerged female with two males in a Petri dish for six hours. Two-day-old females were used in all experiments.

### Age specific developmental time and parasitism *of E. bimaculata*

Small confinement cages were made from transparent reagent bottle caps (2.5 cm diameter), into which a capsule-sized hole had been punched for ventilation. Approximately 15–20 unsexed *B. tabaci* adults were released into each clip cage placed on soy bean leaves. After t24 hours, all adult whiteflies were aspirated from the clip cages and the area of the leaf covered by the clip cages was marked with indelible ink. The egg-infested plants were confined in cages (60 cm × 60 cm × 60 cm) and host stages were allowed to develop to the desired instars (sedentary first, second, third, fourth and pharate adults). When the desired host stage was reached, 40–50 different stage nymphs (sedentary first, second, third, fourth and pharate adults) were independently exposed to 3 mated *E. bimaculata* parasitoids held in a clip cage. *Bemisia tabaci* nymphal instars were identified by measuring the body length of the whitefly, and young instars were selected based on their relatively flat appearance (Gelman et al. 2002b). All whitefly nymphs that were not of the desired instar were removed from the leaf before the parasitoids were released. This was done to ensure that all the exposed instars were of the same age. Clip cages with parasitoids were removed after 24 hours and the soybean leaf was left in the cage to trap emerging adult flies. On each subsequent day, several randomly selected *B. tabaci* nymphs were dissected until the egg, first, second and third instars of *E. bimaculata* were obtained. Parasitoid developmental time was counted from the day the female parasitoid was released. Egg to first instar developmental time was determined when the first instar was observed during dissection. Developmental times for second instar, third instar, prepupa (yellowish-white instar preceding the appearance of black wasp pupa, with two large meconia on either side of the host puparia), and pupa were determined in the same way. For each instar parasitized, at least 10 parasitized whitefly nymphs were dissected each day after parasitization until parasitoid adults emerged. When a wasp pupa was formed, it was removed and placed in a petri dish. The developmental time from egg to pre-pupa, pre-pupa to pupa and pupa to adult *E. bimaculata* eclosion was recorded separately on all whitefly nymphs (sedentary first, second, third, fourth and pharate adults). Parasitoid total developmental time (egg to adult) for each instar was also recorded from the same observations. Percent parasitism was estimated for all *B. tabaci* instars based on the number of nymphs in which parasitoid eggs were found following dissection. Percentage of successful adult parasitoid eclosion were also estimated based on the number of adult parasitoids that eclosed and the total number of whitefly nymphs that were parasitized (which was only an estimate of total parasitism) separately for each host stage of *B. tabaci.* All experiments were repeated six times for each *B. tabaci* host stage.

### Developmental time and parasitism with respect to host plant

Developmental time and parasitism of *E. bimaculata* on the three test plants was achieved using the same procedure as in the age specific and developmental time experiment except that *B. tabaci* third instar was used as host for the parasitoid on the three test plants. Approximately 40–50 unsexed *B. tabaci* adults collected from the culture were released into each clip cage placed on the leaves of the test plants, soybean, cowpea and garden bean. After 24 hours, all adult whiteflies were aspirated from the clip cages and the area of the leaf covered by the clip cage was marked with indelible ink. The egg infested plants were confined in cages (60 cm × 60 cm × 60 cm) and immatures were allowed to develop to the third instar stage which is the most preferred stage for parasitization by *Encarsia* sp.(Donnell and Hunter 2002). When the desired third nymphal stage was reached, 4050 - nymphs were exposed to 3 mated *E. bimaculata* parasitoids held in a clip cage. Clip cages with parasitoids were removed after 24 hours and the plants left in cages. Developmental time, percent parasitism and percentage emergence on the three host plants were recorded as described in the age specific experiment. All experiments were repeated 6 times for each host plant.

### Female adult longevity and fecundity

To determine female fecundity and longevity, a newly emerged *E. bimaculata* female from the same colony was mated and confined by means of a leaf cage (2.5 cm diameter) to the abaxial side of a fully expanded host plant leaf containing between 15–20 whitefly nymphs (third instar). The female was transferred to a fresh leaf every 24 hours until death to determine the fecundity (number of eggs laid by female parasitoids over her lifetime). After each day, the leaves of the test plants with *B. tabaci* nymphs that were exposed to the parasitoid were examined under a dissecting microscope for oviposition. Mortality was registered daily. All the host plants with nymphs that were exposed to the parasitoid were kept in cages until the parasitized nymphs contained wasp pupae. The number of eggs laid in the life time of each female was determined based on the number of wasp pupae formed and from nymphs in which the parasitoid immature died before reaching the wasp pupal stage. Leaves with wasp pupae from each host plant were enclosed in Petri dishes with the top covered with plastic for adult emergence and subsequent sex determination. The parasitoids that emerged from the pupae were counted and sexed as described by Heraty and Polaszek (2000). Survivorship and number of eggs laid each day by the F1 generation were recorded. Fecundity and longevity data were used to calculate daily and lifetime fecundity of *E. bimaculata.* Twenty parasitoids were tested for each host plant with each host plant replicated 6 times.

### Life table and demographic parameters

Life tables were constructed using sex ratio, survivorship, age-specific (the nymphal stage parasitised) fecundity of adults and survivorship and developmental time of all immature stages to calculate intrinsic rate of increase (r_m_), finite rate of increase (λ), net reproductive rate (R_o_), mean generation time (T_c_) and doubling time (T_d_) using the formulae of Andrewartha and Birch (1954).

### Statistical Analysis

Data for developmental time (egg deposition to adult emergence), percentage parasitism, and percentage emergence on different *B. tabaci* instars and the three host plants were analysed using one-way analysis of variance (ANOVA); lifetime fucundity (eggs laid and hatched over the female's lifetime), daily fecundity (eggs laid and hatched per day), and longevity on the three host plants were also analysed using one-way analysis of variance (ANOVA). Means were compared using Fisher's Least Significant (LSD) test at 0.05 level of significance (SAS Institute 2001).

## Results and Discussion

### Developmental time of *E. bimaulata* with respect to *B. tabaci* host age

In a non-choice arena, *E. bimaculata* was capable of parasitizing all nymphal stages of *B. tabaci* and was able to complete development through to the adult stage.

**Table 1.**
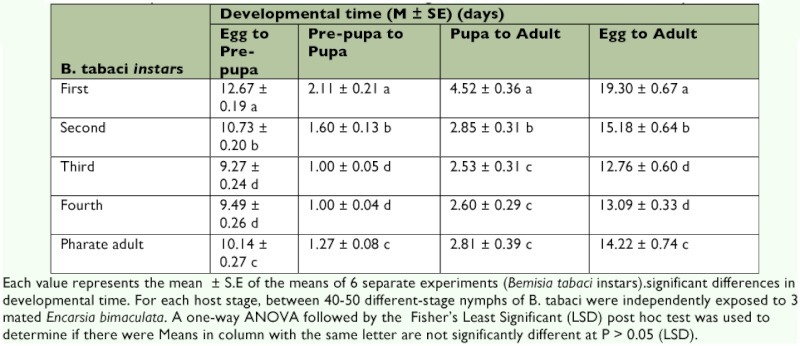
Mean developmental time of *Encarsia bimaculata* attacking different instars of *Bemisia tabaci* on *Glyine max.*

The developmental rate of *E. bimaculata* varied with host age (instar parasitized) (Table 1). The duration of parasitoid development from oviposition to adult emergence was significantly different (F_29,60_ = 139.28, P = 0.0001) longer when the first instars were parasitized than when the second, third, fourth and pharate adults were parasitized (Table 1). There was no significant difference in developmental times when third or fourth instars were parasitised. The durations of individual parasitoid stages were also influenced by host age at the time of parasitization (Table 1). Egg to pre- pupa (F _29,60_ = 290, df = 4 , P = 0.0001), pre-pupa to pupa (F _29,60_ = 140, df = 4 , P = 0.0001) and pupa to adult (F _29,60_ = 55.92, df = 4, P = 0.0001) developmental times were significantly different depending upon host age. Developmental times for these three stages were always significantly longer when first instar whiteflies were provided for oviposition (Table 1). The lengthening of the egg and larvae developmental times in younger *B. tabaci* instars has also been reported for other heteronomous aphelenides (Walter 1983).

However, these stages developed significantly faster when the third or fourth instars were parasitised than when first, second or pharate adults were parasitized (Table 1). Similar findings were reported by Donnell and Hunter (2002); who observed significantly shorter developmental time and more synchronous adult emergence of *E. formosa* when third and fourth instars were parasitized. For egg to adult column (Table 1) values do not appear to be significantly different from those reported by Hu et al. (2002) (standard error overlap) which is surprising considering the reported influence of host plant species and the fact that different wasps were used.

The general preference of *E. bimaculata* for older instars may be related to certain advantages these instars confer to both the ovipositing females and the would-be developing immature. Firstly, the female can assess the host resources exactly with regard to size, quality and suitability at the moment of oviposition. Secondly, eggs laid in the earlier instars will suffer the same age-related mortality risks such as predation and disease. Thirdly, shorter developmental time in the late instars reduces the period for which the offspring is susceptible to hyperparasitism. Nechols and Tauber (1977) reported that wasps that begin development in third and fourth instars nymphs exhibited higher survivorship and developmental times were shortened by approximately 38 %.

**Table 2.**
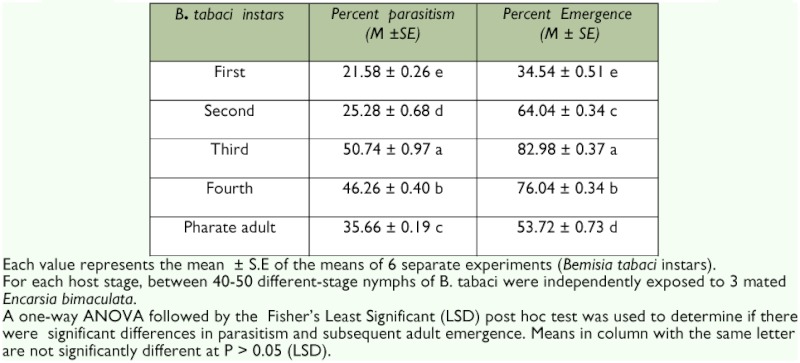
Estimated percent parasitism and emergence of *Encarsia bimaculata* attacking different *Bemisia tabaci* instars on *Glyine max.*

### Percentage parasitism and emergence of *E. bimaculata* with respect to *B. tabaci* host age.

Percent parasitism was also influenced by host age (Table 2). Percent parasitism was significantly (F _29,60_ =3097 df = 4, P = 0.0001) higher when the third or fourth instars of *B. tabaci* were parasitized than when the pharate adults, second or first instars were parasitized (Table 2). The mean percent parasitism across the various instars were not much different from the values reported by Antony *et al.* (2003) for *E. transvena* parasitizing *B. tabaci* instars. These authors observed that even though *E. formosa* oviposited in all *B. tabaci* instars, the third and fourth instars were mostly preferred for oviposition compared to the pharate adults or younger instars. Lopez-Avilla (1988) also reported that *E. formosa, E. luteola, E. adrinae* and *E. cibcenses* parasitized all instars of *B. tabaci* but the third instar had the highest percentage parasitization. Experimental evidence is lacking as to what cues wasps use to determine host size. However, Nell *et al.* (1976) reported that wasp may use their antennae to obtain olfactory and resonance information about hosts, and this information coupled with stimuli received while making 180° turns on the dorsum of the nymph may be used to determine host size.

When *E. bimaculata* parasitized third and fourth instars of *B. tabaci,* successful adult parasitoid emergence was significantly (F _29,60_ = 124.20, df = 4, P = 0.0001) higher when third or fourth instars were parasitised as compared to when first, second or pharate adults were parasitised (Table 2). Similar observations were reported by Antony et al. (2003) for *E. transvena* parasitizing *B. tabaci* instars. Nell et al. (1976) also reported higher emergence when *E. formosa* parasitized third and fourth instars of *T. vaparariorum.* The low emergence of parasitoids in the younger instars of *B. tabaci* could be related to the high percentage of age-related immature mortality.

**Table 3.**
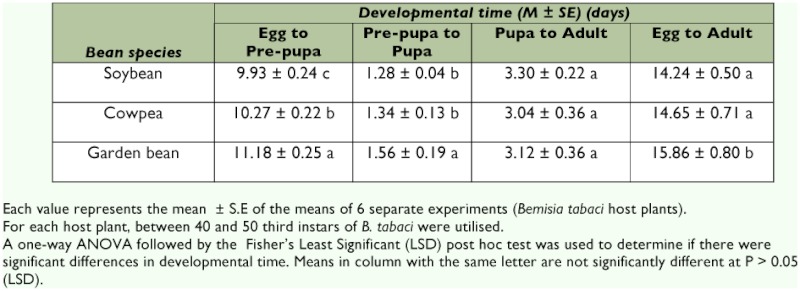
Mean developmental time of Encarsia bimaculata attacking the third instar of Bemisia tabaci on Glycine max, Vigna unguiulata and Phaseolus vulgaris.

### Developmental time *of E. bimaulata* with respect to host plant species

Parasitoid development can be influenced by the nature of plant secondary compounds eaten by their herbivorous hosts (Awmack and Leather 2000). Thus, variation in secondary compounds among individual plants may cause variation in development and successful parasitism of herbivores by specialist parasitoids such as *E. bimaculata.* With respect to the bean species, the duration of parasitoid development on the third instar of *B. tabaci* from oviposition to adult emergence was significantly (F _29,60_ = 45.80, df = 2, P = 0.0002) longer for parasitoids that developed on garden bean compared to cowpea or soybean (Table 3).

The mean developmental time of the parasitoid on cowpea and garden bean were not much different from the range (15–15.80 d) reported by Shishehber (1996) for *E. formosa* at the same temperature. On the other hand, the mean value recorded on soybean was slightly different from the range (16–18d) reported by De Barro et al. (2000) for *E. bimaculata* on soybean under a temperature regime of between 22–30° C. Similarly, Arakawa (1982) reported that *E. formosa* raised on *T. vaporariorum* showed differences in developmental time of between 15 days on tobacco, egg plant, and tomato compared with 24.5 days on poinsettia at a mean temperature of between 22.5-25° C.

### Percentage parasitism and Emergence of *E. bimaculata* with respect to host plant

The role of plants in the interaction between parasitoid and hosts has been known for some time (Price *et al.* 1980; Turling *et al.* 1995). Thus, the host plant species on which the parasitoid develops plays a significant role in determining the level of parasitism in subsequent generations.

Comparatively, more nymphs were parasitized on cowpea followed by garden bean and soybean (Table 4); but, the differences were not significant. Similar observation was reported by Goolsby *et al.* (1998) in which *Encarsia* sp. were found to parasitized more nymphs on rockmelon, cotton and hibiscus than tomato and soybean. The reason for the differential parasitization rates on the three bean species may be related to slight differences in leaf morphology among the bean species particularly between soybean and the other two bean species.The leaves of the soybean species used in the study were covered with hairs (trichomes) which prevent the underside of the whitefly nymphs from fitting level to the surface of the leaf thus making it difficult for *E. bimaculata* to oviposit on the nymphs (Headrick *et al.* 1996). Also, these hairs may interfere with the movement of the parasitoids thereby reducing parasitism (Headrick et al. 1996). This may account for the low parasitism rate on soybean compared to the other species. On the other hand, the leaves of the other two species were relatively smooth, hence the margin of the whitefly nymph's body can fit level with the leaf surface making it easier for the parasitoid to oviposit. The slight difference in percentage parasitism between cowpea and garden bean may be attributed to the comparatively larger veins on garden bean that might interfere with the foraging behaviour of the parasitoid (Van Lenteren *et al.* 1976).

**Table 4.**
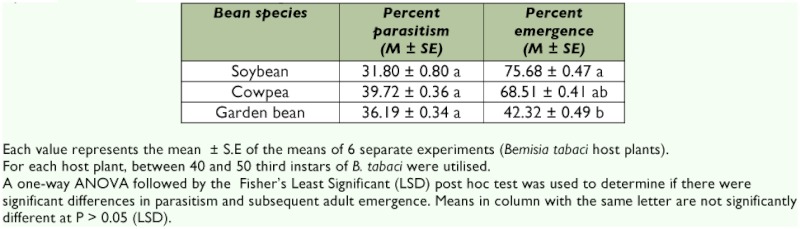
Estimated percent parasitism and emergence of *Encarsia bimaculata* attacking the third instar of *Bemisia tabaci* on *Glyine max, Vigna unguiulata* and *Phaseolus vulgaris.*

When third instars of *B. tabaci* were presented to *E. bimaculata* for oviposition, percent adult parasitoid emergence was significantly (F_29,60_ = 3.96, df = 2, P = 0.0480) different (Table 4). The highest emergence rate was recorded on soybean followed by cowpea and garden bean. This observation is in accordance with studies carried out by Reed *et al.* (1991), which showed that plant species or cultivars identity may influence the proportion of emerging parasitoids. The comparatively higher emergence rate of the parasitoid on soybean and cowpea, respectively, compared to garden bean may be attributed to the high immature mortality of the parasitoid on garden bean. The negative effect of a cultivar exhibiting antibiosis on the pre-imaginal mortality of parasitoids has also been reported by Van Emden (1991) and Farrar *et al.* (1994). It is well-known that secondary substances ingested by phytophagous insects may have negative effects on the biological characteristics (survival, fecundity and development) of parasitoids (Godfray 1994). It has been demonstrated that parasitoids are generally more sensitive to toxic compounds than their polyphagous hosts, as they appear to be incapable of metabolizing the different concentrations of plant secondary compounds present in their host (Turling *et al.* 1995).

**Figure 1.**
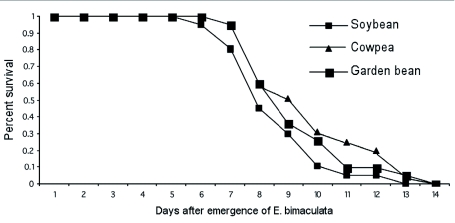
Proportion of female *Encarsia bimaculata* surviving as a measure of longevity on *Bemisia tabaci* infested host plant species. Time of parasitoid emergence and surviving parasitoids were recorded daily on all host plant species. For each host plant, n = 20. The day on which adult parasitoid emergence was first observed was designated day 1 (“onset”) and the corresponding survival was 1 (“100 percent survival”). Percent adult survival for day 1 and for each succeeding day = number of adults that survived on a given day/total number of original cohort that survived. Standard errors have not been indicated, but this value was about 10% of the point's value. High quality figures are available online.

**Table 5.**
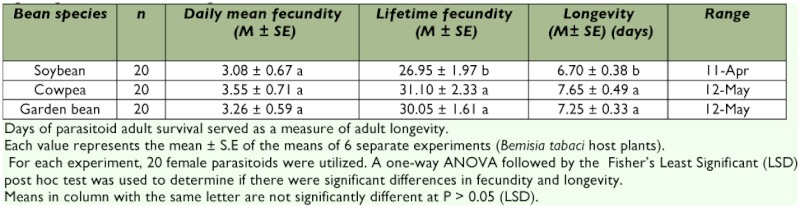
Adult female fecundity and longevity of *Encarsia bimaculata* attacking the third instar of *Bemisia tabaci* on *Glycine max, Vigna unguiulata* and *Phaseolus vulgaris.*

### Adult longevity and fecundity

Adult longevity was recorded as part of the fecundity experiment. Although most female parasitoids died by day 9, 11 and 10 on soybean, cowpea and garden bean, respectively, some females were long lived, surviving to 11 d on soybean and 12 d on cowpea and garden bean (Figure 1).

There were significant (F _29,60_ = 4.15, df = 2, P = 0.0439) differences in the mean longevity of *E. bimaculata* on the three bean species (Table 5). *Encarsia bimaculata* lived longer on cowpea and garden bean as compared to soybean (Table 5).

The mean longevity on the three bean species were lower than the value (10.4 d) reported by Lopez and Botto, (1995) for local populations of *E. Formosa* on tomato. These means were however, higher than the value (5.4 d) reported by Powell and Bellows (1992) at 29° C for an *Eretmocerus* species (likely *E. eremicus*), on *B. tabaci* on cotton. The host plant species we used (soybean, cowpea and garden bean) were different from those used by Lopez and Botto (tomato) and Powell and Bellows (cotton). Also, Powell and Bellows used a different wasp species (*Eretmocerus* sp). A combination of the above factors might have resulted in the discrepancies between our results and those of the other authors. The relatively higher survivorship of the parasitoid on cowpea and garden bean respectively compared to soybean could be attributed to differences in both surface structure and chemistry among the three bean species.

Female *E. bimaculata* laid an average 26.95 eggs during their lifetime on soybean, 31.10 eggs on cowpea and 30.05 eggs on garden bean. These means were within the reported range for other aphelinids (Viggiani 1984). The means were significantly different (F _2,57_ = 45.80, df = 2, P = 0.0002) on the three bean species (Table 5). The daily mean fecundity were however not significantly different (F _4,57_ = 0.3907, df = 2, P = 0.6937) on the three bean species (Table 5).

**Table 6.**
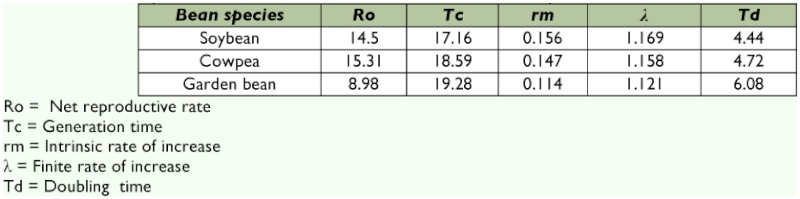
Life table parameters for *Encarsia bimaculata* on the three bean species

### Life table

Results from development and fecundity experiments were used to develop 1x-mx life tables for *E. bimaculata* on the three bean species. These tables were used were used to calculate the demographic parameters shown in table 6. The sex ratio of *E. bimaculata* was 0.7087.

The net reproductive rates (R_o_) of *E. bimaculata* on soybean, cowpea and garden bean are shown in Table 6. The values recorded on soybean and cowpea were higher than that on garden bean. These values for soybean and cowpea were higher than that reported by Powell and Bellows (1992) for wasps attacking *B. tabaci* on cotton (11.74) but, all recorded values were lower than the value (26.50) reported by the same authors for wasps reproducing on cucumber. The low reproductive rate of the parasitod on garden bean indicates that less parasitod females were produced on it compared to soybean and cowpea, respectively.

Generation times (T_o_), the finite rate of increase (λ) and doubling time (T_d_) on soybean, cowpea and garden bean are shown in Table 6. Doubling times were not much different among the bean species but, were lower on soybean followed by cowpea and garden bean. The recorded values for λ on soybean and cowpea were slightly different from garden bean

The most important parameter calculated from the life table is the intrinsic rate of increase, r_m_, which is the rate of increase per individual in an environment where fecundity and survivorship are maximal in the absence of external mortality factors. The larger the r_m_ value, the greater the potential of a species in reproducing and increasing in number within a given environment. This parameter allows for the comparison of r_m_ among species, and it also facilitates the evaluation of a parasitoid concerning its use in strategies where biological control is an option (Jansen and Sabelis 1992). The r_m_ value for soybean, cowpea and garden bean are shown in Table 6. These values are lower than the value (0.28) reported by Lopez and Botto (1995) for *E. formosa.* Small differences in r_m_ values can make remarkable differences in expected population growth over time, therefore, to compare the population growth of the parasitoid on the three bean species over time, the exponential equation for population growth N_t_ = N_o_e^rt^ was used, where N_o_ is the initial number of parasitoid on the plant, Nt is the number of parasitoid at time t, r_m_ the intrinsic rate of increase and t the time in days. Given a stable age distribution, the parasitoid population on soybean, cowpea and garden bean were calculated as 211.43, 236.38 and 81.11, respectively, within two generations.

Given these life table parameters, parasitoid populations should build up relatively slowly on garden bean compared to the other two bean species. Thus, the parasitoid is comparatively a poor biological control agent on garden bean compared to the other bean species. This conclusion however, cannot be applied to all populations of parasitoids on the three bean species used as the effectiveness of a parasitoid as a biological control agent is dependent, among other factors, on the morphological and chemical composition of the host plant and on the identity the parasitoids population.
